# Application of B-ultrasound information image in Renal Puncture Biopsy treatment and Nursing

**DOI:** 10.12669/pjms.37.6-WIT.4831

**Published:** 2021

**Authors:** Linyan Dong, Junhong Li, Mixia Zhao, Jing Ren

**Affiliations:** 1 Linyan Dong, Nurse-in-Charge. Department of Nephrology, Xingtai People’s Hospital, Xingtai 054000, Hebei, China; 2 Junhong Li, Nurse-in-Charge. Department of Nephrology, Xingtai People’s Hospital, Xingtai 054000, Hebei, China; 3 Mixia Zhao, Nurse-in-Charge. Department of Nephrology, Xingtai People’s Hospital, Xingtai 054000, Hebei, China; 4 Jing Ren, Nurse-in-Charge. Department of Nephrology, Xingtai People’s Hospital, Xingtai 054000, Hebei, China

**Keywords:** B-ultrasound, chronic kidney disease, US-PRB, renal puncture biopsy, nursing

## Abstract

**Objectives:**

This study was to explore the application value of B-ultrasound in guiding puncture biopsy of chronic kidney disease (CKD) and the clinical nursing effects under the guidance of B-ultrasound.

**Methods:**

Pathological examination of kidney biopsy was performed on 94 patients with CKD under the guidance of ultrasound from August 2020 to December 2020.; patients were observed for symptoms such as low back pain, backache, hematuria, and subcapsular hematoma. Color Doppler ultrasonography was performed on the punctured patients on day 1, 2, and 3 to observe whether there was subrental hematoma. The pathological results were analyzed and the success rate of percutaneous renal biopsy under ultrasound guidance was analyzed. Before the patient was discharged, investigate the satisfaction with the nursing work.

**Results:**

(1) After the puncture, 45 patients developed low back pain and low back pain symptoms, 12 cases developed subcapsular hematoma; 8 cases showed gross hematuria, 62 cases showed microscopic hematuria, and the rest had no obvious symptoms; (2) the nursing satisfaction rate of 94 cases was as high as 95.7%.

**Conclusion:**

US-PRB is a safe and effective auxiliary examination method, which can improve the success rate of puncture and reduce postoperative complications. Effective nursing can reduce the incidence of postoperative complications and improve patient satisfaction.

## INTRODUCTION

Due to the variety of kidney diseases and the more complicated etiology and pathogenesis, some of the clinical manifestations and pathological changes are not completely consistent. With the rapid development of ultrasound technology in China, it is widely used in various aspects. Ultrasound technology has been applied to percutaneous renal biopsy due to its accurate positioning, high success rate, and few complications.^1^-^4^ Renal puncture is a traumatic examination, it may cause a series of complications such as bleeding and infection after the puncture.^5^ Therefore, carrying out reasonable and effective nursing is of great significance to alleviate the suffering of patients and reduce the occurrence of complications.

This study intended to analyze the clinical effects and nursing methods of percutaneous renal biopsy guided by B-mode ultrasound to provide data support for reasonable care of renal puncture, which is reported as follows.

## METHODS

Gradient normalized mutual information function of B-ultrasound algorithm.^6^-^7^ In this paper, a gradient normalized mutual information function (*I_GNMI_*) is constructed as a similarity measure to increase registration accuracy and stability. Let the reference image and the image to be registered in this paper be A, B, and the normalized mutual information of A and B is


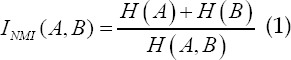


In digital images, gradient can be represented by its first-order partial derivative. Small area templates and image convolution are commonly used for approximate calculations. Sobel edge detection operators are widely used in gradient calculations. The reference images A and B to be registered in this paper are arbitrary. The first-order partial derivative Δ*_x_A(u)*,Δ*_y_A(u)*,Δ*_x_B(u)*,Δ*_y_B(u)* of the pixel u in the x and y directions is calculated by the Sobel edge detection operator. The gradient information between A and B is


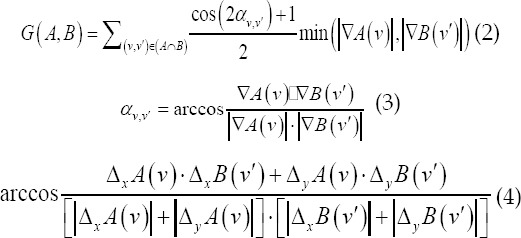


Where *v,v′* is the corresponding point pair where image A and image B coincide, *α_v,v′_* is the gradient vector direction angle between *v,v′*, ∇*A(v)*, ∇*B(v′)* and *v,v′* are the gradient vectors, is the vector point multiplication, and ∣^∇*A(v)*^∣, ∣^∇*B(v′)*^∣ and *v,v′* are the gradient vector amplitudes respectively. To simplify the calculation, the magnitude of the gradient vector is replaced by the sum of the absolute values of the first partial derivatives in the x and y directions.

The gradient normalized mutual information (*I_GNMI_*) between A and B is


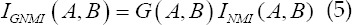


Under the condition that the range of X and Y is [-40,40], with 1 pixel as the step size, the *I_GNMI_* value at each position is calculated separately, as shown in Fig.2 (a), it appears at the registration position Sharp peaks, but there are many local extremes. In formula (1), the denominator H (A, B) is the joint entropy between A and B. When it is far away from the registration position of A and B, H (A, B) gradually becomes larger, and *I_NMI_(A,B)* becomes smaller, which prevents the distance The local extremum at the registration position; however, the numerator is constant and the local extremum is not suppressed. Considering that the mutual information between images decreases rapidly away from the registration position, the numerator is replaced with the mutual information. Therefore, the improved gradient normalized mutual information *(I_NMI_)* is


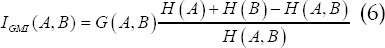


*I_GMI_* integrates gray information and spatial contour information. Its three-dimensional distribution is shown in Fig.2 (b). *I_GMI_* has sharp peaks. The maximum positions of *I_GMI_* and *I_GNMI_* are at x=-8, y=8. Therefore, *I_GMI_* maintains the positioning accuracy of *I_GNMI_*, and the local extreme value is significantly reduced.

Research objects. A total of 94 CKD patients in this group were treated with percutaneous renal biopsy under ultrasound guidance in our department from August 2020 to December 2020. Among them, there were 59 males and 35 females; the age was (36.2±3.57) years. Patients had varying degrees of hematuria, proteinuria, and creatinine in the range of 30.4 to 708.6 μg/L, all of which required a clear diagnosis of renal biopsy. All patients had no contraindications to renal puncture.

Instruments. Using the Siemens Acuson S2000 ultrasound system and Mindray M7 color ultrasound system, the probe selection was 4C1, and the frequency was adjusted to 3.5 MHz; before the ultrasound-guided renal puncture, the patient used two-dimensional ultrasound to check the size, morphology and blood flow of the kidney.

**Fig.1:**
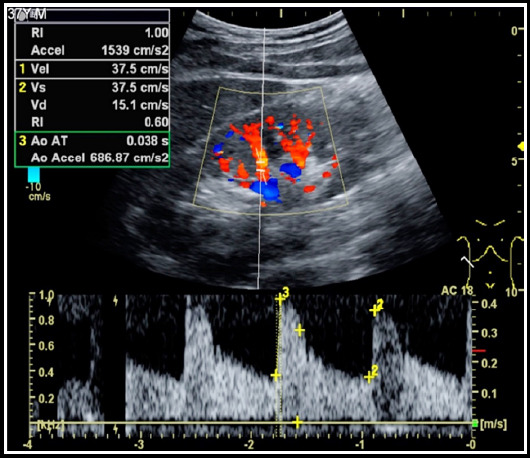
Renal puncture under ultrasound guidance.

Surgery can be performed after all the preliminary work is ready. The range-adjustable rapid automatic biopsy gun is equipped with a dedicated biopsy needle. The patient takes a prone position, fully exposing the puncture kidney area, and a soft cushion on the abdominal pad. Routine disinfection cloth, B-type ultrasound probe positioning, local anesthesia at the puncture point, and then guided by the B-type ultrasound probe, the needle is inserted to the inferior renal capsule, so that the patient pauses breathing at the end of inspiration and quickly press. The biopsy gun button, then the biopsy needle is pulled out, depending on how much tissue is penetrated to determine the number of needle insertions. After taking out the biopsy gun, press the puncture point for about five minutes. The puncture point was disinfected with iodine, covered with sterile gauze, and fixed with adhesive tape. After the operation, lift the patient in parallel to the bed. as shown in Fig.1.

### Preoperative preparation

After the patient is admitted to the hospital, the responsible nurse will ask the patient’s condition and general information again, including whether the patient has a history of bleeding disorders, and complete the relevant examinations according to the doctor’s order before the operation, so as to prevent the patient during the operation and after the operation. Bleeding due to coagulopathy, preoperative examinations mainly include blood routine, urine routine, coagulation four items, renal function, double kidney B-mode ultrasound. The responsible nurse will explain the precautions during and after the operation, and ask the doctor to order Anticoagulant and vasodilator drugs were discontinued three days before surgery.

The patient’s vital signs should be closely monitored after surgery to detect and control the occurrence of complications at any time. Vital signs monitoring including breathing, pulse, blood pressure, urine output, urine color.^8^-^10^

### A. Psychological Intervention

because renal puncture is an invasive examination, patients will experience pain or bleeding symptoms after surgery. Therefore, nursing staff should adopt narrative nursing, emotional counseling, and other skills to communicate with patients. They can be distracted by music, broadcasting, or watching videos to relieve their pain. In addition, patients should be informed about the importance of an optimistic attitude.

### B. Condition Care

a. the caregiver pressurized the affected area of the patient for 15 minutes after the operation, sterilized the puncture needle with iodine tincture, and covered it with sterile gauze. The waist was pressed with a sandbag for six hours. b. the patient slept on a rigid board bed for 6 hours in a supine position. During this period, they were forbidden to violently turn over or raise the buttocks. If there was no hematuria after 24hour, they can get out of bed. c. The caregiver should monitor the patient’s blood pressure once every 6 hours. After the indicators were stable, it was checked twice a day. At the same time, the body temperature should be checked every 4 hours. If the body temperature rose, it should be checked whether there was infection. d. the patient’s urine routines were detected 3 times consecutively, factoring into the color, urine output, bleeding, etc. The hematuria indicated prolong bed rest, and immediate treatment is needed.

### C. Complication Care

a. hematuria: after renal puncture, the hemorrhage usually disappears within three days. The patient should be instructed to stay in bed, and take hemostatic drugs on time and in the amount. They should drink 600mL water within two hours to reduce the probability of blood clot formation in the ureter or kidney. For patients with renal insufficiency, the caregiver should instruct them to drink an appropriate amount of water and monitor the urine dynamically; ***Urine retention:*** if urinary retention occurred, the vagina should be washed with warm water. If it was ineffective, sterile indwelling catheterization was required. It was removed the next day and the patient was allowed to urinate on his own. c. ***Perirenal hematoma***: if the patient’s main complaints involved pain in the waist and abdomen, and the test found tenderness at the puncture site and local bulge, then it was considered hematoma. Patients should be instructed to rest, take medications, and have blood transfusions if necessary. After 48h, warm compress on the waist was needed.

### Observation indicators

the observations included the occurrence of complications and patients’ satisfaction with renal biopsy under the guidance of B-ultrasound, as shown in Table-I.

**Table-I:** The satisfaction degree grading.

*Satisfaction degree*	*Score*
Satisfied	80-100
Relatively satisfied	60-80
General	Lower than 60

## RESULTS

Ninty four patients who underwent US-PRB, all the materials were successfully obtained. The length of the material taken was 10 to 18 mm, the length of the puncture was >12 mm, and the number of glomeruli under the light microscope was> 10. The length of the puncture was one to three times. In terms of the puncture length: 22 cases were successfully punctured with one time length, and the puncture success rate was 23.4% (22/94); 53 cases were successfully punctured with 2 times length, and the success rate was 56.4% (53/94); 19 cases were successfully punctured with 3 times length, and the success rate was 20.2% (19/94).

(2) The patient was supine for 24 hours after the puncture, and the patient was observed. 45 (47.9%) patients reported symptoms of low back pain and backache; 62 (66%) patients had microscopic hematuria, 8 (8.5%) patients had gross hematuria, and the remaining patients had no obvious symptoms of discomfort; Patients underwent bedside color Doppler ultrasonography on days one, two and three after puncture, and found that 12 patients (12.8%) had subcapsular hematomas on the first day, and the size of the hematomas did not increase significantly within 3 days or Decrease. Regular follow-up of the subcapsular hematoma showed that the hematoma disappeared fastest 35 days and the hematoma disappeared 68 days.

(3) Of the 94 patients, 32 had IgA nephropathy, 20 had membranous nephropathy, 13 had lupus nephritis, 9 had mesangial proliferative glomerulonephritis, 8 had glomerulosclerosis, and diabetic nephropathy. There were six cases, three cases of membranous proliferative glomerulopathy, one case of purpuric nephritis, one case of multiple myeloma nephropathy, and one case of amyloidosis nephropathy.

Among 94 patients, 78 patients were satisfied with the clinical nursing, 12 were relatively satisfied, and four were generally satisfied, so the postoperative nursing satisfaction of patients was as high as 95.7%.

## DISCUSSION

### Significance of renal biopsy

Defining pathological classification: clinically multiple nephrotic syndromes, there can be multiple types of pathology, the treatment effect and disease outcome are different.^11^ Kidney biopsy is the gold standard for clinical diagnosis of kidney diseases. After the pathological type is determined by kidney biopsy, a reasonable treatment plan can be formulated. Some experts have proposed that, renal puncture biopsy can intuitively detect changes in the glomerulus and the presence or absence of immune complexes, suggesting prognosis and outcome.^12^,^13^ Studies have found that, after renal biopsy, the correction rate of clinical diagnosis is 34% to 63%, and the correction rate of treatment plan is 19% to 36%.^14^

### The advantages of ultrasound-guided renal puncture

During the operation, the renal puncture technique guided by B-ultrasound can observe the condition of the puncture needle in the kidney from multiple levels and angles, which can further reduce the damage to the large blood vessels and surrounding tissue in the kidney, and is safe.^15^,^16^ Relevant studies have shown that compared with CT-guided renal biopsy, this method is safe, economical, less traumatic, shorter in recovery period, and has strong operability and reproducibility.^17^-^19^

###  Nursing care for complications

B ultrasound-guided percutaneous renal biopsy is a traumatic examination. Studies have shown that the operation can cause various complications, especially bleeding, and good nursing methods can effectively reduce complications.^20^,^21^ Therefore, nursing methods are important. Results in a study of 94 patients showed that 45 (47.9%) had symptoms of low back pain and back pain; 62 cases (66%) had microscopic hematuria, 8 cases (8.5%) had gross hematuria, and the remaining patients had no obvious symptoms, but the above complications didn’t appear after special treatment. one case of local hematoma capsule was cured. Studies have also proposed that, post-examination care can not only reduce the incidence of complications, such as infection, low back pain, and gross hematuria, but also ensures the success rate of kidney biopsy guided by B ultrasound.^22^ After investigation, the patient’s satisfaction with the care before and after the puncture reached 95.7%, which further illustrates the necessity of the nursing method under the guidance of B-ultrasound.

US-PRBis invasive, it is easy to operate and less risky. The position of the puncture needle can be observed in real time on the ultrasound screen, thereby improving the success rate of puncture, and is therefore widely used in clinical practice]. In this study, the success rate of renal biopsy was 100%, but the range of 1 to 3 punctures was needed. The reason may be that the patient failed to cooperate with the operator to perform the puncture. In this study, all puncture patients chose to puncture the right kidney because the right kidney is lower than the left kidney and is easy to operate. The lower pole of the right kidney should be selected during operation, try to let the puncture needle puncture the kidney tissue in the parenchyma of the lower pole, and avoid the puncture needle from entering the collection system to avoid causing hematuria. This is consistent with the report that the puncture site should be selected under the kidney. This place has the most renal cortex, the highest success rate of material extraction, and it is not easy to damage the kidney, reducing the possibility of hematuria.

## CONCLUSIONS

Renal puncture under ultrasound guidance is widely used in clinical practice, and has important clinical significance for the diagnosis, treatment and prognosis of kidney diseases. Studies have shown that percutaneous renal biopsy is performed under the guidance of ultrasound, which greatly improves the accuracy of the needle insertion point, ensures the adequacy of material extraction, and greatly improves the safety. Therefore, the diagnostic value of percutaneous renal biopsy guided by B-mode ultrasound is clear, and it is worthy of clinical promotion. During the puncture process, effective care should be given to help improve clinical efficacy. In summary, Kidney biopsy guided by ultrasound is a safe, has high-success rate, and auxiliary examination method is of great significance for clinical diagnosis and treatment.

### Authors Contribution:

**LD:** Conceived the study, literature review, participated in its design, analyzed the data and helped to draft the manuscript and also the responsible and accountable for the accuracy or integrity of the work. **JL & MZ:** Helped in design, data collection, article drafting & critical revision.**JR:** Takes the responsibility and is accountable for all aspects of the work in ensuring that questions related to the accuracy or integrity of any part of the work are appropriately investigated and resolved.
